# Computational Design of the Affinity and Specificity of a Therapeutic T Cell Receptor

**DOI:** 10.1371/journal.pcbi.1003478

**Published:** 2014-02-13

**Authors:** Brian G. Pierce, Lance M. Hellman, Moushumi Hossain, Nishant K. Singh, Craig W. Vander Kooi, Zhiping Weng, Brian M. Baker

**Affiliations:** 1 Program in Bioinformatics and Integrative Biology, University of Massachusetts Medical School, Worcester, Massachusetts, United States of America; 2 Department of Chemistry and Biochemistry, University of Notre Dame, Notre Dame, Indiana, United States of America; 3 Department of Molecular and Cellular Biochemistry, University of Kentucky, Lexington, Kentucky, United States of America; 4 Harper Cancer Research Institute, University of Notre Dame, Notre Dame, Indiana, United States of America; Fox Chase Cancer Center, United States of America

## Abstract

T cell receptors (TCRs) are key to antigen-specific immunity and are increasingly being explored as therapeutics, most visibly in cancer immunotherapy. As TCRs typically possess only low-to-moderate affinity for their peptide/MHC (pMHC) ligands, there is a recognized need to develop affinity-enhanced TCR variants. Previous in vitro engineering efforts have yielded remarkable improvements in TCR affinity, yet concerns exist about the maintenance of peptide specificity and the biological impacts of ultra-high affinity. As opposed to in vitro engineering, computational design can directly address these issues, in theory permitting the rational control of peptide specificity together with relatively controlled increments in affinity. Here we explored the efficacy of computational design with the clinically relevant TCR DMF5, which recognizes nonameric and decameric epitopes from the melanoma-associated Melan-A/MART-1 protein presented by the class I MHC HLA-A2. We tested multiple mutations selected by flexible and rigid modeling protocols, assessed impacts on affinity and specificity, and utilized the data to examine and improve algorithmic performance. We identified multiple mutations that improved binding affinity, and characterized the structure, affinity, and binding kinetics of a previously reported double mutant that exhibits an impressive 400-fold affinity improvement for the decameric pMHC ligand without detectable binding to non-cognate ligands. The structure of this high affinity mutant indicated very little conformational consequences and emphasized the high fidelity of our modeling procedure. Overall, our work showcases the capability of computational design to generate TCRs with improved pMHC affinities while explicitly accounting for peptide specificity, as well as its potential for generating TCRs with customized antigen targeting capabilities.

## Author Summary

T cell receptors (TCRs) play a major role in immunity, recognizing peptide antigens presented by major histocompatibility complex proteins. Due to their capacity to target intracellularly produced proteins and initiate cell killing, there is significant interest developing TCR-based therapeutic strategies, particularly towards cancer. A concern with TCRs is their weak-to-moderate affinities, which limits therapeutic potential. While in vitro evolution has been used to enhance TCR affinity, with sometimes spectacular results, these techniques can reduce peptide specificity and offer little control over affinity enhancements. Here we explored the use of structure-based computational design to enhance TCR affinity, which in principle can permit control over both specificity and affinity gains. We examined a clinically relevant TCR recently used in melanoma immunotherapy, identifying and characterizing mutations which enhanced affinity with no detectable impacts on binding specificity. We solved a crystal structure of our highest affinity designed TCR in complex with antigen, which indicated high accuracy of the structural modeling during the design process, and we critically evaluated several design protocols and functions to further improve design success. These results provide valuable insights into the use of computational design for TCRs. Lastly, the enhanced affinity variants identified may be of potential clinical benefit.

## Introduction

T cell receptors (TCRs) are key elements of adaptive immunity, as they specifically recognize antigenic peptides bound to MHC proteins (pMHCs) on cell surfaces and are responsible for initiating immune responses against targeted cells. The TCR-pMHC interaction is of considerable importance in health and disease, notably in transplantation, autoimmunity, and is a target for development of vaccines and therapeutics for infectious disease and cancer [1]–[3]. For example, the adoptive transfer of genetically engineered T cells, whereby tumor-specific TCRs are transduced into T cells and then infused into the patient, is being explored as a means for cancer immunotherapy. Clinical trials of such genetically engineered T cells have shown promise in the treatment metastatic melanoma [4]–[6] and synovial cell carcinoma [7], leading to durable tumor regression and long-term survival in patients.

The observations that TCRs have relatively weak affinities towards pMHC (typically 1–300 µM; ∼1000-fold lower than mature antibody/antigen interactions) and that pMHC affinities are correlated to some extent with *in vivo* potency [8] have led to a number of efforts to engineer TCRs with enhanced binding affinity. These efforts include *in vitro* selection [9]–[13] as well as computational structure-based design [14]–[16], resulting in up to 1,000,000-fold improvements in affinity. However, a major concern in enhancing TCR affinity is maintenance of peptide specificity. As TCRs recognize peptides presented by MHC proteins, yet invariably form contacts to both peptide and MHC [17], enhancements to TCR affinity risk dangerous cross-reactivity if affinity-enhancing substitutions preferentially target the MHC protein. Such “off-target” interactions can be challenging to predict from peptide sequence and are a major concern for high affinity TCRs [18]. Indeed, the unanticipated cross-reactivity of a high affinity TCR resulted in serious consequences and deaths in a recent clinical trial [19]. Additionally, significant enhancements in antigen-specific affinity may be detrimental for T cell activity, as there is evidence of a TCR “threshold affinity” above which T cell responsiveness is attenuated [20], [21]. Thus, careful control of affinity and specificity is crucial in the development of enhanced TCRs for therapeutic purposes.

The αβ TCR DMF5 was originally isolated from tumor infiltrating lymphocytes present in a patient with metastatic melanoma [22]. DMF5 recognizes the 27–35 nonameric and 26–35 decameric peptide epitopes from the MART-1 melanoma antigen presented by the class I MHC protein HLA-A*0201 (HLA-A2), and was the second TCR to be used in clinical trials of genetically engineered T cells [5]. Without knowledge of structure or affinity, Robbins and colleagues previously examined a series of point mutations in DMF5, generating variants that resulted in improved antigen-specific responses yet also showed evidence of reduced specificity, underscoring the need for incorporating structural information in the design process [23]. More recently, the DMF5 TCR has been crystallized by our laboratory in complex with both the MART-1 nonameric epitope (AAGIGILTV; referred to as AAG) as well as the anchor-modified decameric epitope (ELAGIGILTV; referred to as ELA), both bound to HLA-A2 [24]. The structures show that despite the significant difference in peptide conformation between the ELA/HLA-A2 and AAG/HLA-A2 ligands [24], DMF5 engages them with an identical binding mode. These structures along with associated affinity measurements provide an ideal opportunity to explore the applicability of computational structure-based design for rationally enhancing a clinically relevant TCR, while simultaneously exploring the impact on peptide specificity.

Utilizing a refined algorithm initially developed for our redesign of the A6 TCR [14], we applied structure-based design to the DMF5 TCR, generating variants and characterizing mutants with affinity enhancements of up to 400-fold toward ELA/HLA-A2. Highlighting the ability of structure-based design to directly target regions of interest within protein interfaces, and in contrast with results seen with *in vitro* selection, the strongest affinity enhancement was achieved with only two previously identified amino acid substitutions [25] that directly interact with the peptide. Importantly, the highest affinity variant showed no detectable recognition of unrelated peptides presented by HLA-A2. We determined the crystallographic structure of this variant bound to ELA/HLA-A2, permitting a detailed analysis of the accuracy of the various structural modeling protocols, and together with the affinity measurements, a quantitative assessment of scoring functions and terms. Further, by purposely disrupting interactions with the ELA peptide, we were able to shift TCR specificity away from the ELA peptide toward the AAG peptide, albeit with more modest efficacy. Altogether, these results highlight the promise of structure-based design for TCR engineering, and provide a rich dataset for further improvements in design strategies, including the broadening of efforts to other TCR-pMHC systems. Lastly, given the ongoing use of the DMF5 TCR in efforts to develop immunological therapies for melanoma (e.g., [26]), the high affinity DMF5 variants identified here may have future clinical applicability.

## Results

### Design and Affinities of DMF5 Point Mutants

**Figure 1:**
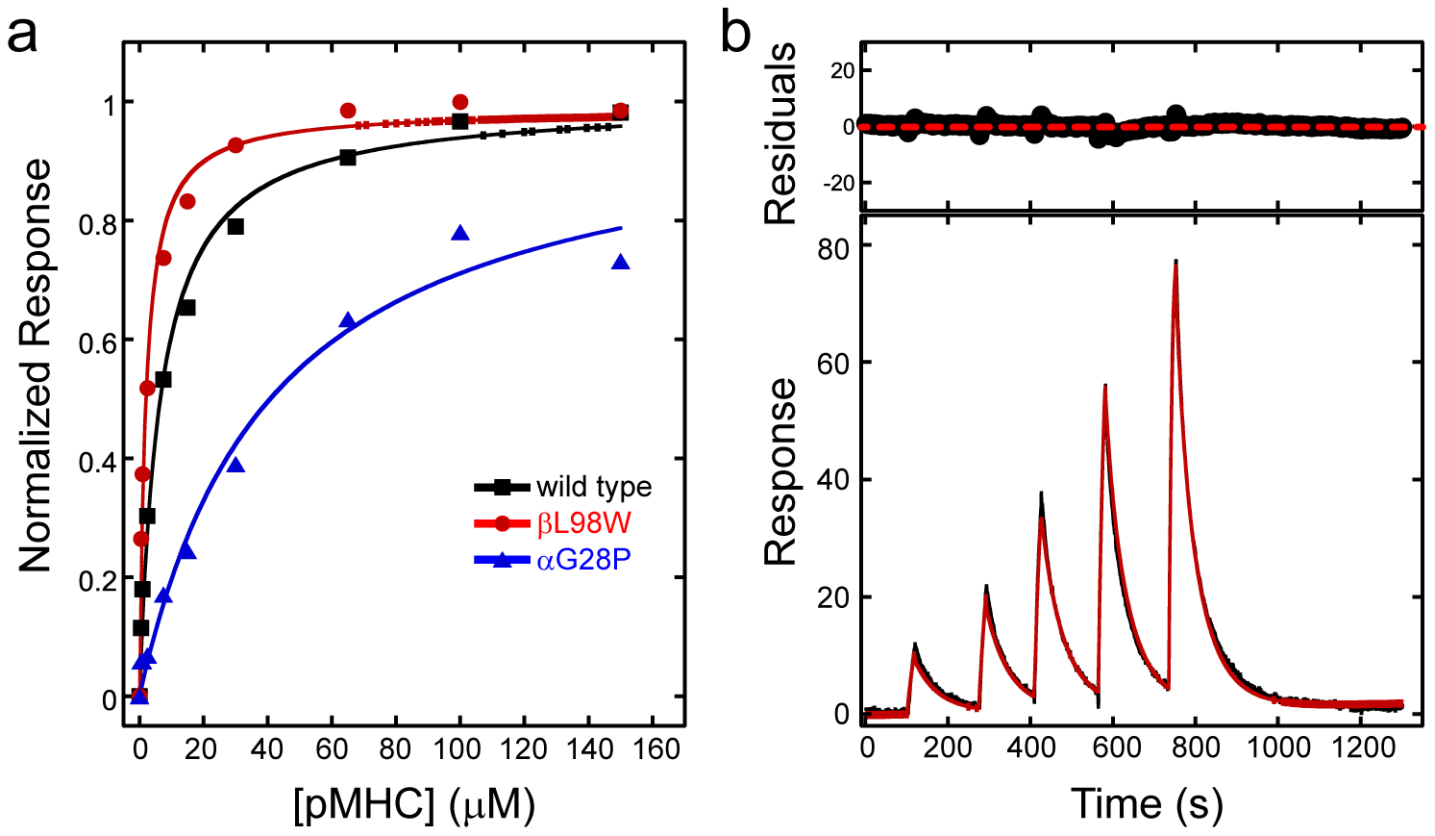
Representative binding affinity measurements. (a) Steady-state binding equilibrium data for ELA/HLA-A2 binding wild type DMF5 and the βL98W and αG28P mutants. Solid lines represent a fit to a 1∶1 equilibrium binding model. (b) Kinetic titration data for ELA/HLA-A2 binding of the high affinity YW (αD26Y/βL98W) mutant of DMF5. Data are in black in the bottom panel; the red line is a fit to a 1∶1 kinetic titration model with drift. Residuals (difference between data and fitted curve) are shown in the smaller top panel.

We used the ZAFFI and Rosetta software tools [14], [27] to predict the affinity changes of DMF5 mutants for ELA/HLA-A2 or AAG/HLA-A2, simulating all point mutations for each DMF5 residue within 5.5 Å of the pMHC ligand in the tertiary structures. In total, we examined 589 substitutions of 31 DMF5 residues within each complex, which were then ranked based on predicted TCR-pMHC affinity. Twelve computationally designed mutations were chosen for experimental testing. To help maintain peptide specificity, with the exception of two αR27 mutants, we only chose mutants that were predicted to contact the peptides. The αR27 mutants were selected to compare with our previously designed substitutions at the corresponding position in the A6 TCR [14], which shares the germline α chain gene (TRAV 12-2) and some MHC contacts with DMF5. We performed mutagenesis using soluble DMF5 gene constructs, expressed and purified the mutant proteins, and measured their binding affinities toward ELA/HLA-A2 and AAG/HLA-A2 via surface plasmon resonance (Figure 1). The mutations and their measured affinities for ELA/HLA-A2 and AAG/HLA-A2 are given in Table 1, organized by the method through which they were selected: Affinity, Specificity, or Proline, as discussed in detail below. In addition, Table 1 includes four mutations, listed under “Test”, that we selected for measurement based on manual inspection of the TCR-pMHC structures.

**Table 1:** DMF5 mutants organized by design strategy and measured affinities toward ELA/HLA-A2 and AAG/HLA-A2.

	ELA				AAG				
Mutant	K_D_, µM	ΔΔG, kcal/mol	ΔΔG Error	Fold Change^1^	K_D_, µM	ΔΔG, kcal/mol	ΔΔG Error	Fold Change^1^	Spec Change^2^
wild-type	9.5	-	-	-	43	-	-	-	-
**Affinity**									
αD26W	**0.68**	**−1.6**	**0.1**	**14**	**1.1**	**−2.2**	**0.1**	**42**	2.9
αD26Y	**0.46**	**−1.8**	**0.1**	**21**	**4.5**	**−1.4**	**0.1**	**10**	0.5
αR27W	26	0.6	0.1	0.4	62	0.2	0.1	0.7	1.9
βL98W	**2.9**	**−0.7**	**0.1**	**3.3**	**11**	**−0.8**	**0.1**	**3.9**	1.1
βF100W	46	0.9	0.1	0.2	83	0.4	0.1	0.5	2.5
βT102F	8.9	−0.04	0.1	1.1	27	−0.3	0.2	1.6	1.5
**Specificity**									
αG28I	41	0.9	0.1	0.2	36	−0.1	0.1	1.2	**5.2**
αG28L	99	1.4	0.1	0.1	130	0.7	0.1	0.3	**3.4**
αG28Y	120	1.5	0.1	0.1	110	0.5	0.1	0.4	**5.2**
**Proline**									
αR27P	12	0.2	0.1	0.8	46	0.02	0.1	1.0	1.2
αG28P	60	1.1	0.2	0.2	340	1.2	0.2	0.1	0.8
βA55P	8.5	−0.1	0.1	1.1	61	0.2	0.1	0.7	0.6
**Test**									
αG28N	40	0.9	0.1	0.2	86	0.4	0.2	0.5	2.1
αY50A	NB	-	-	-	NB	-	-	-	-
αG94T	NB	-	-	-	NB	-	-	-	-
βF100Y	100	1.4	0.1	0.1	160	0.8	0.1	0.3	2.8
**Combinations**									
αD26W/βL98W	**0.033**	**−3.3**	**0.1**	**290**	**0.60**	**−2.6**	**0.1**	**72**	0.2
αD26Y/βL98W	**0.024**	**−3.5**	**0.1**	**400**	**1.7**	**−1.9**	**0.1**	**30**	0.1

**Bold** denotes measured affinity improvements, or specificity changes, greater than 3-fold.

1 Improvement in binding association constant relative to wild-type (K_D__mut/K_D__wt).

2 Specificity change toward AAG versus ELA peptide: Fold_Change AAG/Fold_Change_ELA.

Mutations in the Affinity category were chosen on the basis of predicted enhancement in affinity towards both ELA/HLA-A2 and AAG/HLA-A2. Three of the six mutations in this category had significantly improved affinities: αD26W, αD26Y, and βL98W. The two αD26 mutants had the highest measured binding affinities among all tested mutants (up to 40-fold improvement for αD26W towards AAG/HLA-A2), while the βL98W mutant had a 3-fold affinity improvement for both ELA/HLA-A2 and AAG/HLA-A2.

**Figure 2:**
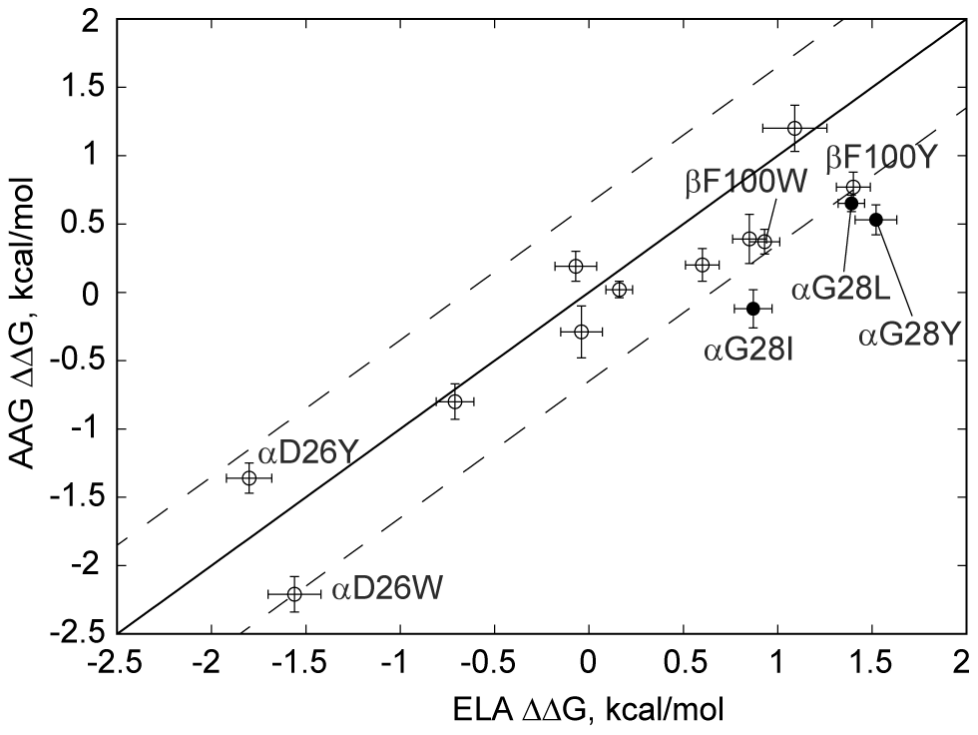
ΔΔG (in kcal/mol) for DMF5 point mutants for nonameric (AAG) versus decameric (ELA) peptide bound to HLA-A2. Solid line denotes equal ΔΔG values, while dashed lines denote a 4-fold affinity shift (0.82 kcal/mol) toward AAG (bottom dashed line) or ELA (top dashed line). AAG and ELA ΔΔG error bars are shown for each mutant, while solid points are the αG28 substitutions selected to shift preference toward the nonameric variant.

Mutations in the Specificity category were chosen on the basis of predicted differential affinity towards ELA/HLA-A2 and AAG/HLA-A2. These were predicted to contact a portion of the interface that varies between the two peptides, where the alanine at the N-terminus of the nonamer is replaced by a larger glutamate residue in the decamer (Figure S1). Several mutations at TCR position αG28 were chosen that would potentially destabilize the interaction with ELA/HLA-A2 via steric hindrance while favoring AAG/HLA-A2. Of the specificity-altering substitutions, all shifted specificity toward the AAG nonamer as predicted, albeit the shifts were relatively modest (up to a 5-fold shift; Table 1 and Figure 2).

Based on work with the A6 TCR [28], as well as the observation of proline CDR mutants in high affinity TCR selection experiments [9], [11], we tested three proline mutations that were predicted to stabilize CDR loops in the bound conformation while not negatively impacting contacts with the pMHC (Proline category in Table 1). None of these proline substitutions showed a significant improvement in affinity, indicating that while potentially reducing the entropic cost for binding, the magnitudes of any such improvements were not substantial enough to yield a net increase in binding free energy, possibly because these loops appear relatively rigid in the unbound DMF5 TCR [29]. Moreover, given the >1 kcal/mol loss in binding free energy with both ELA/HLA-A2 and AAG/HLA-A2, the αG28P substitution may have directly or indirectly impacted pMHC contacts, consistent with its relatively buried position in the pMHC interface.

Combining the affinity-enhancing αD26Y and βL98W mutations (this double mutant is referred to as YW) yielded a substantial improvement towards ELA/HLA-A2. This high affinity double mutant was previously described in a brief report, with a preliminary affinity measurement yielding an approximate 200-fold enhancement [25]. Here, however, we measured a 400-fold improvement (from 9.5 µM to 24 nM). The difference is attributable to our use of a kinetic titration binding assay in this case (Figure 1b), which is more accurate at quantifying binding in the nanomolar range or higher, as it permits analyses of high affinity binders without requiring surface regeneration [30]. The on and off rates of the YW mutant towards ELA/HLA-A2 determined from the kinetic titration were 1.7×10^6^ M^−1^ s^−1^ and 0.05 s^−1^, respectively. The dissociation rate of wild type DMF5 from ELA/HLA-A2 was too fast to accurately measure [29], indicating that the combined mutations result in a slower TCR off rate, as seen with the majority of affinity-enhanced TCRs [31].

The combined YW mutations were somewhat nonadditive (−3.5 kcal/mol enhancement versus −2.5 kcal/mol assuming additivity), suggesting a modest degree of communication between the CDR1α and CDR3β loops; the same degree of cooperativity was also observed for the αD26W/βL98W (WW) mutant binding ELA/HLA-A2 (Table 1). Nonadditivity within TCR binding interfaces has been observed previously [32], [33], and could be attributable to structural or dynamic effects of mutations on neighboring loops. The YW variant also showed a smaller but still considerable 30-fold enhancement towards AAG/HLA-A2. The reduced affinity enhancement is likely attributable to the lack of the N-terminal glutamate in the AAG peptide as discussed below.

Given its dramatic affinity improvement toward both ELA/HLA-A2 and AAG/HLA-A2, we next asked whether the high affinity YW variant could recognize targets other than the MART-1 nonamer and decamer. No binding was detectable towards HLA-A2 presenting the Tax or gp100 peptides, even at concentrations more than 25-fold higher than those used to characterize binding to wild type DMF5 (Figure S2). The Tax and gp100 peptides have markedly different sequences from ELA or AAG (Tax: LLFGYPVYV; gp100: IMDQVPFSV), yet the conformations of HLA-A2 are identical in the four peptide/HLA-A2 crystal structures [24], [34], [35]. The lack of detectable binding of the high affinity DMF5 YW variant towards the other peptides thus suggests that we may have improved its specificity towards the MART-1 peptides, and at the minimum demonstrates that our design has avoided peptide-independent targeting of HLA-A2.

### Comparison with Predicted Affinities

**Figure 3:**
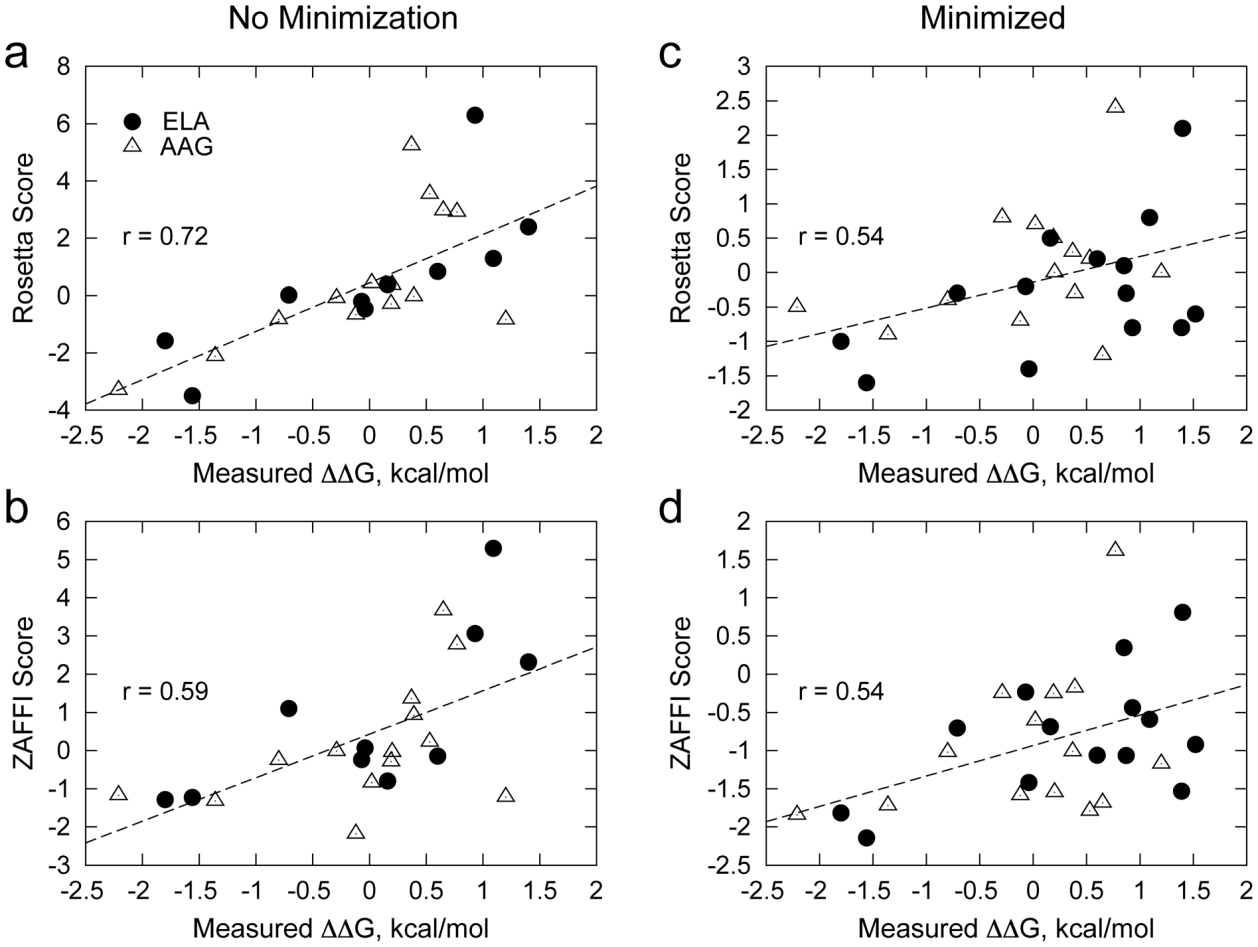
Predicted versus measured ΔΔGs for measured DMF5 point mutants binding to ELA/HLA-A2 (solid circles) and AAG/HLA-A2 (empty triangles), using the Rosetta (a, c) and ZAFFI (b, d) functions. Mutations were modeled in Rosetta without minimization (a, b) or with minimization of interface backbone and side chains (c, d). For (a) and (b), four outlier points with poor measured ELA/HLA-A2 binding and highly unfavorable scores are not shown. For each plot, best fit lines and correlations (all calculated without the four outlier points for consistency) are given.

To quantify the performance of the design methods that we used to generate candidate mutations, ZAFFI and Rosetta, we compared predicted versus measured affinities towards ELA/HLA-A2 and AAG/HLA-A2 for each of the point mutations that were experimentally characterized (excluding the αY50A and αG94T mutants, for which binding was too weak to measure). Mutants were scored with or without structural minimization (referred to as Min and NoMin respectively), as shown in Figure 3 (with scores in Table 2). For both the Rosetta and ZAFFI scoring functions, the NoMin simulations yielded higher agreement with experimental data (Figure 3a–b), with the Rosetta scoring function achieving an impressive 0.72 correlation with measured ΔΔGs (excluding four outlier points correctly predicted to have poor affinities). Except for the proline mutant αG28P, the Rosetta NoMin protocol made no other false positive predictions, and its top four predictions (αD26Y and αD26W for the two pMHCs) had the highest measured affinities among all predicted point mutations (βL98W was also correctly ranked highly, particularly for AAG). This predictive success is notable as the majority (8 out of 14) of these mutants involved glycine and proline, which are often overlooked during in silico studies due to difficulties predicting backbone-related effects [27].

**Table 2:** Scores from ZAFFI (ZF) and Rosetta (Ros), predicted peptide contacts, and measured energies (in kcal/mol) for DMF5 point mutants binding to ELA/HLA-A2 and AAG/HLA-A2.

	ELA						AAG					
Mutant	ZF	Ros	ZFMin	RosMin	Pep Conts^1^	ΔΔG	ZF	Ros	ZFMin	RosMin	Pep Conts^1^	ΔΔG
αD26W	**−1.23**	**−3.50**	**−2.14**	**−1.60**	E1	**−1.56**	**−1.17**	**−3.30**	**−1.84**	−0.50		**−2.21**
αD26Y	**−1.28**	**−1.58**	**−1.82**	**−1.00**	E1	**−1.8**	**−1.32**	**−2.11**	**−1.72**	**−0.90**	A1	**−1.36**
αR27P	**−0.80**	0.38	**−0.69**	0.50		0.16	**−0.84**	0.43	**−0.61**	0.70		0.02
αR27W	−0.18	0.83	**−1.06**	0.20		0.6	−0.04	0.37	**−1.55**	**0.00**		0.2
αG28I	15.83	8.01	**−1.06**	−0.30	E1	0.87	**−2.18**	**−0.67**	**−1.59**	**−0.70**	A1	−0.12
αG28L	38.09	29.33	**−1.53**	**−0.80**	E1	1.39	3.66	2.97	**−1.68**	**−1.20**	A1,A2	0.65
αG28N	9.50	5.00	0.34	0.10	E1	0.85	0.93	−0.03	−0.18	−0.30	A1,A2	0.39
αG28P	5.30	1.29	−0.59	0.80	E1	1.09	**−1.22**	**−0.84**	**−1.17**	0.00	A1	1.2
αG28Y	9.21	4.38	**−0.92**	**−0.60**	E1	1.52	0.23	3.55	**−1.79**	0.20	A1	0.53
αY50A	1.70	1.50	1.63	1.50	I5	>2.0	1.02	0.75	0.95	0.80		>2.0
αG94T	1.49	1.94	0.57	1.50		>2.0	6.89	4.83	1.36	3.40		>2.0
βA55P	−0.29	−0.20	−0.23	−0.20		−0.07	−0.29	−0.29	−0.25	0.50		0.19
βL98W	1.10	0.02	**−0.70**	−0.30	L8	**−0.71**	−0.25	**−0.83**	**−1.02**	−0.40	L7,T8	**−0.8**
βF100W	3.06	6.30	−0.44	**−0.80**	A3,G4^2^	0.93	1.33	4.96	**−1.01**	0.30	I4,I6,T8	0.37
βF100Y	2.33	2.39	0.81	2.10	G4	1.4	2.80	2.92	1.61	2.40	I4	0.77
βT102F	−0.06	−0.46	**−1.42**	**−1.40**	I5	−0.04	−0.01	−0.13	−0.25	0.80		**−0.29**

Scores were produced using fixed backbone and fixed neighboring side chains (ZF, Ros) or minimization of interface backbone and side chains (ZFMin, RosMin) of wild-type and mutant structures. **Bold** denotes measured ΔΔG better than −0.25 kcal/mol, or prediction score of ≤−0.6 for ZF or Ros, which we found to correspond to −0.25 kcal/mol based on fitting to 26 measured point mutations of the A6 TCR.

1 Peptide residues within 6.0 Å of the predicted mutant side chain (modeled without minimization).

2 Additional peptide residue contacts were predicted in the structural model; the two closest are shown.

The ZAFFI NoMin protocol gave a correlation of 0.59 with measured data (again excluding several true negative outlier points due to predicted steric hindrance). Though it previously outperformed Rosetta in scoring A6 TCR mutants [14], and correctly gave favorable scores for the DMF5 αD26 mutants, ZAFFI made several false positive DMF5 predictions for both AAG/HLA-A2 and ELA/HLA-A2, possibly due to its parameterization on a more limited dataset than Rosetta and the distinct biophysical properties of the A6 and DMF5 interfaces. This led us to evaluate and reparameterize the terms in the ZAFFI function using a larger set of energy terms and mutants, as described further below.

Both minimization-based protocols (ZAFFI Min and Rosetta Min; Figure 3c–d), while displaying positive correlations with the experimental results, were lower in their predictive success than the NoMin protocols. However, ZAFFI Min scored the αD26 mutants favorably, and correctly identified βL98W (for AAG) as within the score cutoff for predicted binding improvement (≤−0.6; for ELA, βL98W was near this cutoff). Overall though, false positive predictions for ZAFFI and Rosetta led to relatively weak correlations, suggesting that minimization may have led to incorrect structures in some cases. We additionally tested other minimization protocols as well as more extensive side chain packing (Table S1), each of which gave lower correlations with measured energies than the relatively restrictive NoMin protocol.

### Crystal Structure of Mutant DMF5 YW in Complex with ELA/HLA-A2

**Figure 4:**
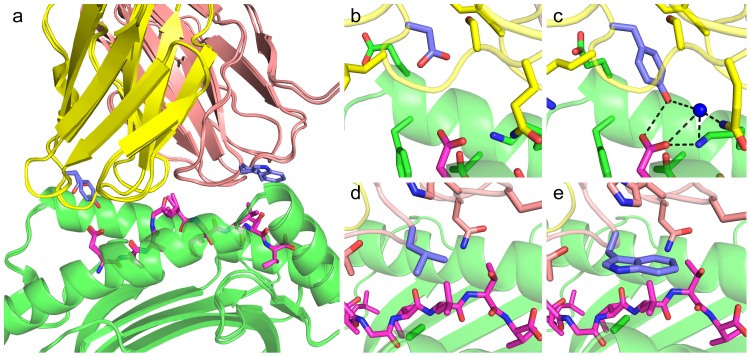
Structure of the DMF5 YW double mutant in complex with ELA/HLA-A2. (a) Superposition of the YW/ELA/HLA-A2 and the DMF5/ELA/HLA-A2 complexes. DMF5 α chain is yellow, β chain is tan, peptide is magenta (shown as sticks), MHC is green, and β2m is cyan; residues that were mutated are shown as sticks. Close-ups of (b) wild-type αD26, (c) mutant αY26, (d) wild-type βL98, (e) mutant βW98 are shown. In (b–e), residues proximal to the mutation sites are shown as sticks, and in (c) hydrogen bonds involving the αY26 side chain and a bound water molecule are shown as dashed lines.

To examine the structural basis of the 400-fold binding affinity improvement and compare with the models generated during the design process, we crystallized and determined the structure of the DMF5 YW mutant bound to ELA/HLA-A2 at 2.56 Å resolution (Figure 4, with crystallographic data in Table S2). Clear electron density was observed for the TCR-pMHC interface, and the positions of the mutated amino acids were unambiguous as indicated by an unbiased, iterative-build OMIT map [36] (Figure S3). As with other structurally characterized TCRs engineered for high pMHC affinity [12], [13], [37]–[39], the docking orientation was conserved when compared to the wild-type complex, with a TCR-pMHC crossing angle of 32°, versus 33° for the wild-type. Essentially no perturbations of the interface CDR loops or peptide were observed (0.34 Å backbone atom RMSD for TCR and pMHC residues within 10 Å of the binding interface), indicating that our relatively conservative design strategy of selecting point substitutions against a fixed pMHC structure did not substantially alter the interface or proximal side chains (Figure 4b–e). This tight structural conservation of the binding loops and target pMHC residues is in contrast to some high affinity TCRs generated by *in vitro* selection where moderate (1G4 designs c5c1, c48c50, c58c61, c58c62) [12],[37] or pronounced (2C designs m6, m13, m67, and Mel5 design α24β17) [13], [38], [39] perturbations of CDR loops were exhibited, along with adjacent CDR loop remodeling [39] and addition of a synergistic ion adduct in the interface [12]. In the recently described structure of the c134 TCR [39] which is an in vitro selected variant of the A6 TCR with nearly 1000-fold improved affinity for Tax/HLA-A2, the mutant CDR3β loop retained largely the same backbone structure as the wild-type loop, yet it led to a shifted footprint of the α chain over the pMHC.

As anticipated from our modeling, both the tyrosine and tryptophan mutant side chains directly contact the MART-1 peptide in the αD26Y/βL98W structure, and make more extensive peptide contacts than their wild-type counterparts (Table S3). These mutations led to a 5% increase in buried solvent accessible surface area for the pMHC, from 1059 Å^2^ to 1113 Å^2^. Unexpectedly, as explicit water molecules were not used in our structural modeling or scoring, a water-mediated hydrogen bond to the peptide was introduced between the mutant residue αY26 and the side chain of the N-terminal glutamate of the peptide, in addition to a direct hydrogen bond between side chains (Figure 4c). This polar network may explain the superior affinity of αY26 versus αW26 for ELA/HLA-A2, despite the fact that they were predicted to be similar (ZAFFI) or αW26 was preferred (Rosetta; Table 2). In contrast, αD26W binds more strongly than αD26Y to AAG/HLA-A2, which lacks the N-terminal peptide glutamate and hydrogen bonding capability at that side chain. In light of the water-mediated contacts observed in the mutant crystal structure, we re-ran simulations using explicit water molecules from the wild-type and mutant structures, but no improvement in correlation was observed (Table S1).

### Evaluation of Modeled Mutant Residues

**Figure 5:**
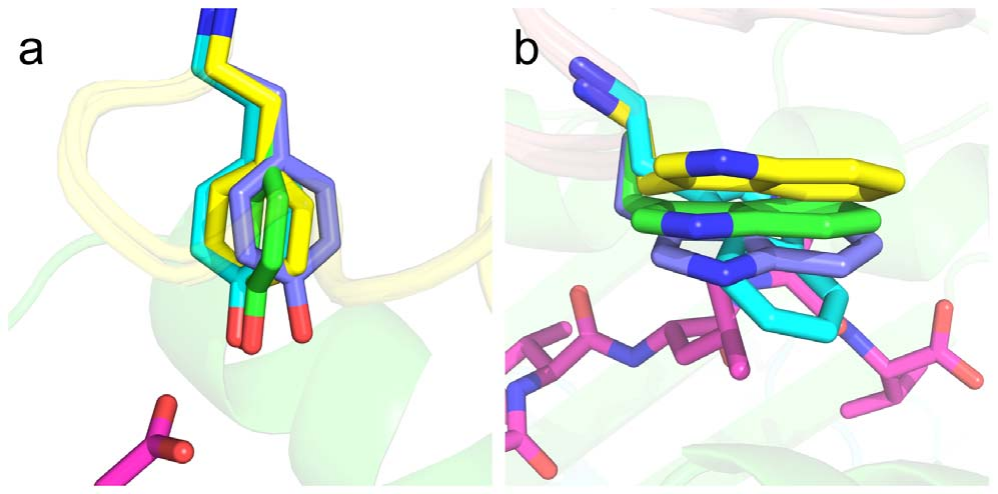
Predicted structures of mutant residues (a) αD26Y and (b) βL98W compared with the crystal structure. Colors for α chain, β chain, peptide, MHC, and mutant side chains from the crystal structure are as in Figure 4. Mutant side chains are shown as sticks, with models in yellow (no minimization), cyan (with minimization), and green (no minimization, in the context of the mutant crystal structure). For simplicity, only the pMHC from the crystal structure is shown.

The crystal structure of the YW variant bound to ELA/HLA-A2 allowed us to evaluate the performance of several structural modeling protocols. After least squares fitting of the backbone of the TCR and pMHC interface residues to the crystal structure, we compared positions of the modeled side chains to those in the crystal structure (Figure 5, with RMSDs in Table 3). In addition to the NoMin and Min methods, we evaluated models generated using two intermediate minimization methods: MinSC (minimizing interface side chains only) and MinBB (minimizing interface backbone atoms). Finally, we re-modeled the engineered side chains in the context of the mutant crystal structure (NoMinMut) to determine whether accurately positioned backbone and neighboring side chain atoms could improve modeling results.

**Table 3:** Root mean square distances (in Å) between predicted mutant residues and those from the crystal structure.

Protocol^1^	αD26Y	βL98W
NoMin	1.06	1.52
MinSC	1.07	1.21
MinBB	1.6	2.26
Min	1.28	2.23
NoMinMut	1.13 (0.98)	0.89

1 Modeling protocol used in Rosetta; NoMin denotes packing only the mutant side chain without minimization while MinSC, MinBB, and Min denote minimization of interface side chains, backbone atoms, or both, respectively. NoMinMut utilized the NoMin protocol, starting with coordinates from the mutant YW-ELA/HLA-A2 crystal structure (with mutant side chains removed prior to modeling). For αD26Y, the value in parentheses is the RMSD with water molecules from the YW-ELA/HLA-A2 structure included in the simulation.

For modeling the side chain of αY26, all protocols performed well in predicting the general orientation of the Tyr side chain, with NoMin outperforming the other protocols (RMSD = 1.06 Å). Though generally accurate, all models exhibited a rotation in the aromatic ring and a slight shift in the OH group with respect to the crystal structure. As these errors were possibly due to the absence of explicit waters in the modeling omitting the water mediated hydrogen bonding observed in the YW/ELA/HLA-A2 crystal structure, we re-ran the NoMinMut simulation with water molecules from that structure, but found little improvement in RMSD (0.98 Å, versus 1.13 Å without water molecules).

The predicted side chain conformations for the mutant βL98W were more variable than αY26, including a flip of the aromatic rings in the Min and MinBB models, leading to relatively high RMSDs (>2 Å) relative to the experimentally determined structure for this residue. The structure modeled without minimization had a sub-optimal positioning of the Trp side chain (tilted away from the pMHC) (Figure 5), which improved substantially (from 1.52 Å to 0.89 Å RMSD) when modeled in the context of the backbone and side chains from the mutant crystal structure. This indicates that Rosetta's packing protocol is sensitive to small structural perturbations and accurate modeling of backbone and neighboring side chains can lead to improved predictions.

### Evaluation of Scoring Terms and Functions for Affinity Prediction

In light of the lower accuracy of the ZAFFI scoring function on the measured DMF5 point mutants (Figure 3) than for the A6 TCR, we performed a systematic evaluation of scoring functions to better predict DMF5 affinities while still maintaining accuracy with the set of A6 mutants. We included several statistical potentials in addition to the energetic and knowledge-based terms from the original ZAFFI study [14]. Given that minimization yielded false positive results for both ZAFFI and Rosetta functions (Figure 3) and that unminimized structures more closely matched the YW-ELA/HLA-A2 crystal structure, we used unminimized models for this analysis. In addition to correlation with measured ΔΔGs, we evaluated scoring functions using receiver operating characteristic area under the curve (AUC) in order to judge discrimination of binding improvement without penalizing true negative or true positive outliers.

We identified a scoring function (referred to as ZAFFI 1.1) with a higher correlation (0.74) than ZAFFI (0.59) and Rosetta (0.72) for the set of DMF5 point mutants (excluding the four αG28 outlier mutants), and high AUC values for both DMF5 and A6 mutants (Table 4 and Figure S4). Correlation P-values are included in Table 4 for all functions, highlighting significant predictive performance of ZAFFI 1.1 (p<0.001) for both sets of data. ZAFFI 1.1 includes six terms: van der Waals attractive and repulsive components, desolvation, intra-residue clash, hydrogen bonding and Coulombic electrostatics. While its correlation with A6 TCR data (0.65) was not as high as the original ZAFFI function (0.77), both the correlation and AUC are considerably higher than Rosetta on that set of data. Although a few outlier points persisted, including αG28P in the AAG/HLA-A2 interface, the overall success of this function demonstrates that a relatively simple scoring function and packing scheme can be used to model a large proportion of energetic changes in three designed TCR-pMHC interfaces. To examine the performance of this function in the context of other protein-protein interactions, we applied it to two large sets of interface point mutants (285 mutants each) of two proteins designed de novo to target influenza hemagglutinin (Table S4), recently used in a collaborative effort to evaluate protein design algorithms as part of the protein docking experiment CAPRI [40]. We found that ZAFFI 1.1 (with NoMin packing) performed similarly to the other tested functions for scoring the HB36 mutants (r = 0.36; p = 2.1×10^−10^), while for HB80 mutants it outperformed all other functions (r = 0.5; p<2.2×10^−16^), with a Kendall tau rank correlation (0.38) higher than we achieved in the CAPRI experiment using a ZAFFI-related function (0.31), where our Kendall correlation surpassed all other groups [40].

**Table 4:** Correlation and ROC AUC values of tested energy functions and structural modeling methods for DMF5 and A6 TCR point mutants.

Function	Packing	DMF5 Corr^1^	DMF5 AUC^2^	A6 Corr^3^	A6 AUC^3^
Rosetta	NoMin	**0.72 (<10^−4^)**	**0.95**	0.42 (<10^−1^)	0.79
ZAFFI	NoMin	0.59 (<10^−2^)	0.86	**0.77 (<10^−5^)**	**0.92**
Rosetta	Min	0.54 (<10^−2^)	0.82	0.52 (<10^−2^)	0.75
ZAFFI	Min	0.54 (<10^−2^)	0.77	0.56 (<10^−2^)	0.79
ZAFFI 1.1	NoMin	**0.73 (<10^−4^)**	**0.93**	**0.63 (<10^−3^)**	**0.92**

1 24 points with DDG values, excluding four (true negative) outliers.

2 All 32 tested ELA and AAG mutants.

3 26 previously measured A6 TCR point mutants.

Values in parentheses are correlation p-values.

Values in bold indicate correlation >0.6, p-value<10^−3^, or AUC>0.9.

## Discussion

Structure-based design of TCRs provides a means to improve upon low wild-type affinities for pMHC while maintaining, improving, or altering specificities for desired targeting capabilities. While some studies have determined the fine specificities of designed TCRs using biophysical [39], [41] and cell-based [23] methods, here we demonstrated that point substitutions selected using structure-based methods can be used to efficiently engineer pMHC specificity and affinity. We then utilized structural modeling and x-ray crystallography to gain atomic-level insights into these substitutions. We achieved higher affinity improvements than previously reported in structure-based TCR design, with just two point substitutions resulting in an approximately 400-fold affinity improvement, versus 150-fold for four combined point mutants of the BC1 TCR selected using molecular mechanics [16], and 100-fold for four combined point mutants of the A6 TCR selected using ZAFFI [14].

Despite the structural plasticity commonly observed in TCR-pMHC interfaces [42]–[45], our computational modeling and crystal structure indicate that carefully selected point substitutions can improve pMHC affinity and modulate peptide specificity without grossly perturbing the interface structure. We note though that a broad extension this approach to other TCRs of interest will likely entail further refinement of the energy function based on measured data, in addition to improvements in high-resolution modeling of TCR-pMHC complexes [46]. Large-scale datasets of mutant binding affinities, including the CAPRI data we utilized to assess our design functions [40], can provide possible training sets for re-weighting terms and derivation of energy-based statistical potentials that would add discriminating power and predictive breadth to the ZAFFI function. Additionally, our analysis of the YW-ELA/HLA-A2 structure indicates that there is room for improving structural modeling of mutant residues, with modeling of fine structural effects and bound water molecules representing two avenues for further development.

The modulation of nonamer versus decamer specificity by many point mutants of the DMF5 TCR highlights the sensitive nature of TCR-antigen recognition, as well as the potential to fine-tune TCR recognition properties via structure-based design. We achieved a shift in specificity toward the nonameric MART-1 peptide via mutation of αG28 residues that were predicted to clash with the decameric E1 residue but would be accommodated in the cleft near the nonameric A1, similar in concept to the “knob-in-to-hole” designs utilized to alter binding specificity in other protein-protein interfaces [47]. The clash with the decamer was overestimated using the NoMin modeling methods (which had the greatest overall predictive success), thus leading to lower than anticipated specificity shifts; better modeling of clashes through judicious use of minimization (avoiding false positive predictions as we observed) could potentially reduce such errors. In contrast, we found an increase in specificity (>4-fold) toward the decameric peptide with the DMF5 double mutants YW and WW, resulting from the cooperativity of these mutants in the presence of the decamer. This peptide-dependent cooperative effect is a previously undescribed mechanism for shifting TCR specificity. As the structure of the YW/ELA/HLA-A2 complex did not suggest any major alterations in the binding interface compared to the wild-type complex, this effect may be dynamic in nature. As recently reported, the Mel5 TCR mutant α24β17, which targets ELA/HLA-A2 with a 30,000-fold affinity improvement over wild-type, was found to retain peptide specificity, albeit towards alanine substituted ELA variants rather than between the ELA and AAG decameric/nonameric peptides [13]. In this case specificity was mediated through subtle solvent interactions. By modeling solvent and dynamic effects, as well as exploring explicit specificity design methods, such as multi-state design [48], greater control of TCR specificity could be achieved via rational engineering.

Three of the α chain mutants we tested were previously examined in the A6 TCR (αD26W, αG28I, and αG28L) [14], whose CDR1α and CDR2α loops are identical to DMF5 due to the common use of the TRAV12-2 gene. αD26W improved pMHC affinity significantly for both TCRs, though to varying extents. On the other hand, the αG28 mutants improved the affinity of A6 modestly (∼2-fold) but resulted in no change or weakened affinity with DMF5. This behavior likely follows from the positions of the mutations, as the αG28 mutants are predicted to make extensive contacts with the varying N-terminus of the peptide, while αD26W would primarily target the same HLA-A2 site to improve affinities for all three pMHCs. However, both αD26 mutants of DMF5 still exhibited a measurable peptide dependence with ΔΔG, compared with, for instance, βL98W which had identical effects in the context of both MART-1 peptides. Data from more mutants and positions, as well as other TCR-pMHC systems, such as the Mel5 TCR which shares the TRAV12-2 gene with DMF5 and A6 and also targets ELA/HLA-A2 with a similar docking mode [49], would help to further delineate the extent of any conserved effects of affinity-enhancing or destabilizing mutants. Indeed, the structure of the high affinity α24β17 Mel5 TCR mutant in complex with ELA/HLA-A2 [13] features a large hydrophobic substitution at position αD26 (Phe), which closely matches the αD26Y conformation and the pMHC binding site in the YW/ELA/HLA-A2 structure (Figure S5), although as Mel5 α24β17 contained 18 additional substitutions, the energetic effect of αD26F alone is unclear. A more detailed study of the impact of affinity-enhancing mutations in germline CDRs would help to further probe TCR germline binding permissiveness suggested by a recent double mutant cycle deconstruction of the interface with the A6 TCR [50].

In conclusion, we have shown that rational, computational-based design offers the potential to simultaneously alter the efficacy and antigen targeting of a therapeutic TCR, potentially enabling the development of improved TCRs for adoptive cell therapy [51] or biotherapeutics [52] customized to bind antigens presented by tumors or virally infected cells from individual patients. Given the ongoing use of the DMF5 TCR in clinical trials for cancer immunotherapy, the higher-affinity YW variant of DMF5 generated here may also be of potential clinical benefit.

## Methods

### Simulation and Scoring of DMF5 Point Mutations

As with our previous study designing the A6 TCR [14], we used the “interface” mode of Rosetta 2.0.2 [27] to model point mutations of the DMF5 TCR. Command line options were specified to include extra chi1, chi2, and chi3 rotamers (“-extrachi_cutoff 1 -ex1 -ex2 -ex3”). Only the mutant side chain was repacked (the default behavior of this mode) while the protein backbone from the wild-type structure was retained. Rosetta predicted mutant structures as well as ΔΔGs, and the structures were then re-scored by our energetic scoring function ZAFFI to generate its own set of predicted ΔΔG scores. The ZAFFI filter, parameterized using the A6 TCR data and designed to remove false positive predictions that destabilized native electrostatic contacts, was not used in this study, given that our focus was evaluation and development of binding energy prediction functions, and the new system and protocols being explored would require tuning of the parameters of this filter. However, the filter function was used to corroborate avoidance of mutations in some cases (such as hydrophobic mutants of αQ30) where key hydrogen bonds would likely be disrupted.

To generate predictions of point mutants using side chain and/or backbone minimization we used Rosetta 2.3, a more recent version of this program that includes minimization functionality in its interface mutagenesis mode. Minimization was specified using the command line flags (“-min_interface -int_bb -int_chi”) to perform minimization of interface backbone and side chain atoms in the wild type and mutant structures (“Min” protocol), while just “-int_chi” or “-int_bb” was used to perform only side chain or backbone minimization (“MinChi”, “MinBB”). Point mutant simulations with explicit water molecules taken from the input structure were also performed using Rosetta 2.3, using the command line flag: “-read_hetero_h2o”.

### Selection of Proline Mutants

We analyzed residue backbone conformations in the bound and unbound DMF5 TCR structures using a Ramachandran plot analysis server [53] (http://zlab.bu.edu/rama/). DMF5 CDR positions with favorable backbone conformations for proline (as well as favorable pre-proline conformations for the preceding residue), in addition to either improved or maintained pMHC affinity predicted for the proline mutant by at least one prediction method, were selected for experimental mutation to proline.

### Protein Expression and Purification

Expression and refolding of soluble constructs of DMF5 TCRs and HLA-A2 were performed as previously described [29], [54]. In brief, the TCR α- and β-chains, the HLA-A2 heavy chain, and β2-microglobulin (β2m) were generated in *Escherichia coli* as inclusion bodies, which were isolated and denatured in 8 M urea. TCR α- and β-chains were diluted in TCR refolding buffer (50 mM Tris (pH 8), 2 mM EDTA, 2.5 M urea, 9.6 mM cysteamine, 5.5 mM cystamine, 0.2 mM PMSF) at a 1∶1 ratio. HLA-A2 and β2m were diluted in MHC refolding buffer (100 mM Tris (pH 8), 2 mM EDTA, 400 mM L-arginine, 6.3 mM cysteamine, 3.7 mM cystamine, 0.2 mM PMSF) at a 1∶1 ratio in the presence of excess peptide. TCR and pMHC complexes were incubated for 24 h at 4°C. Afterward, complexes were desalted by dialysis at 4°C and room temperature respectively, then purified by anion exchange followed by size-exclusion chromatography. Refolded protein absorptions at 280 nm were measured spectroscopically and concentrations determined with appropriate extinction coefficients. Mutations in the DMF5 α- and β-chains were generated by PCR mutagenesis and confirmed by sequencing. Peptides and plasmids were commercially synthesized and purified (Genscript).

### Surface Plasmon Resonance

Surface plasmon resonance experiments were performed with a Biacore 3000 instrument using CM5 sensor chips. In all experiments, TCR was immobilized to the sensor chip via standard amine coupling and pMHC complex was injected as analyte. All samples were thoroughly dialyzed in HBS-EP buffer (20 mM HEPES (pH 7.4), 150 mM NaCl, 0.005% Nonidet P-20), then degassed for at least 15 minutes prior to use. Steady-state experiments were performed with TCRs coupled onto the sensor chip at 1000–1500 response units. Injections of pMHC spanned a concentration range of 0.5–150 µM at flow rates of 5 µl/min at 25°C. Multiple data sets were globally fit using a 1∶1 Langmuir binding model utilizing BIAevaluation 4.1. Kinetic titration experiments were performed with TCRs coupled at approximately 500 response units. A series of five ELA titrations, spanning 10–160 nM and 20–320 nM at 2-fold increase per titration, were flowed over YW and WW respectively. Flow rates of 30 µl/min were used at 25°C. Data were fit with a 1∶1 association model with drift using BIAevaluation [30].

### Crystallization, Diffraction Data Collection, Structural Refinement and Analysis

Crystals of the DMF5 YW-ELA/HLA-A2 complexes were grown from 12% PEG 3350, 0.25 M MgCl_2_ buffered with 0.1 M HEPES (pH 8.0) at 25°C. Crystallization was performed using sitting drop/vapor diffusion. For cryoprotection, crystals were transferred into 20% glycerol/80% mother liquor for 30 s and immediately frozen in liquid nitrogen. Diffraction data were collected at the 22ID (SER-CAT) beamlines at the Advanced Photon Source, Argonne National Laboratories. Data reduction was performed with HKL2000. The ternary complexes were solved by molecular replacement using PHENIX and Protein Data Bank (PDB) entry 3QDG as the reference model [29]. Rigid body refinement, followed by translation/libration/screw (TLS) refinement and multiple steps of restrained refinement were performed. TLS groups were automatically chosen by phenix.refine. Once defined, TLS parameters were included in all subsequent steps of the refinement. Anisotropic and bulk solvent corrections were taken into account throughout refinement. After TLS refinement, it was possible to unambiguously trace the position of peptides and TCR CDR loops in all structures against σ_A_-weighted 2F_o_-F_c_ maps. Evaluation of models and fitting to maps were performed using COOT [55]. The template structure check in WHATIF [56] and MolProbity [57] was used to evaluate the structures during and after refinement. Atomic positioning was verified with an iterative-build OMIT map calculated in PHENIX [36]. Structures were visualized using PyMOL [58]. Analysis of hydrogen bonds was performed with HBPlus [59], using hydrogen-acceptor maximum distance of 2.7 Å and a donor-acceptor maximum distance of 3.6 Å. Solvent accessible surface areas were measured in Discovery Studio (Accelrys Inc.) using a probe radius of 1.4 Å. The structure has been deposited with the Protein Data Bank (PDB ID 4L3E).

### Analysis and Retraining Scoring of Affinities

ROC AUC analysis was performed using the CROC package [60]. Multi-linear regression to determine weighting of terms was performed as described previously, using 760 measured point mutants from four enzyme-inhibitor complexes [14]. However, we used van der Waals attractive and repulsive terms from Rosetta [27] rather than the corresponding terms from ZRANK [61], as the former led to some improvement in performance across the tested systems. As with the original ZAFFI training, we removed mutants with high clash during training (van der Waals repulsive score >580, corresponding to 48 mutants removed out of 760). We included a number of statistical potential terms for evaluation that were recently tested for binding affinity prediction [62], though none led to substantial improvements in predictive performance in this context. The terms and weights for the retrained energy function (ZAFFI 1.1) are:

van der Waals attractive: 0.57

van der Waals repulsive: 0.0045

solvation: 0.58

hydrogen bonding: 1.2

intra-residue repulsion: 0.026

electrostatics: 0.03

Solvation, hydrogen bonding, and intra-residue repulsion terms were obtained from Rosetta (along with the van der Waals terms as noted above), while the electrostatics term is the long-range Coulombic electrostatics energy from ZRANK [61].

### Correlations

All correlations (with the exception of the Kendall tau rank correlations reported in Table S4) are Pearson correlations. P-values for correlations were calculated using the program R (www.r-project.org).

## Supporting Information

Figure S1 Structural variability of nonameric (AAG; cyan) and decameric (ELA; magenta) MART-1 peptides bound to wild-type DMF5 (from wild-type complex structures, PDB IDs 3QDJ and 3QDG). MHC and TCR colors are as in Figure 4; DMF5 residue αG28 is shown as spheres for reference.
(PDF)

Figure S2 The high affinity DMF5 variants show no recognition of the Tax_11–19_ or gp100_209(2M)-217_ peptide/HLA-A2 complexes. a) Injections over a wild-type DMF5 surface. The main response shows injections of MART-1_26(27L)-35_/HLA-A2, with the binding response indicated. The inset shows injections of gp100/HLA-A2 and Tax/HLA-A2 over the same surface, with no response at concentrations as high as 400 mM. b) Injections over a high affinity YW DMF5 surface. Injected pMHC is as in panel a. c) Injections over a high affinity WW DMF5 surface. Injected pMHC is as in panel a.
(PDF)

Figure S3 Electron density for βW98 (gold) and αY26 (purple) in the YW-ELA/HLA-A2 crystal structure contoured at 1σ calculated from an unbiased, iterative-build OMIT map. The density shows the clear, unambiguous positioning of the two mutated residues.
(PDF)

Figure S4 Predictions from ZAFFI 1.1 compared with measured ΔΔGs for DMF5 point mutants binding to ELA/HLA-A2 (solid circles) and AAG/HLA-A2 (empty triangles). Best fit line and correlation are given; the four true negative outlier points omitted from Figure 3 are omitted here as well.
(PDF)

Figure S5 Comparison of mutant TCR αD26Y residue in the YW-ELA/HLA-A2 complex with the corresponding mutant position (αD27F) in the α24β17-ELA/HLA-A2 complex. Complexes were superposed by fitting pMHC backbone atoms. The mutant αD27F is shown in orange sticks, ELA peptide from α24β17-ELA/HLA-A2 in pink sticks, and all other colors are as in Figure 4.
(PDF)

Table S1 DMF5 mutant predictive performance for additional tested packing protocols.
(PDF)

Table S2 X-ray data collection and refinement statistics for the crystal structure of the DMF5 αD26Y/βL98W - ELA/HLA-A2 complex.
(PDF)

Table S3 Contacts between mutant DMF5 residues and ELA/HLA-A2.
(PDF)

Table S4 Correlations with measured values and corresponding p-values for HB36 and HB80 mutants.
(PDF)
