# Supplementary figures and images for: Computational Design of the Affinity and Specificity of a Therapeutic T Cell Receptor
Source: PLoS Comput Biol. 2014 Feb 13;10(2):e1003478. doi: 10.1371/journal.pcbi.1003478 (PMC3923660; doi:10.1371/journal.pcbi.1003478)

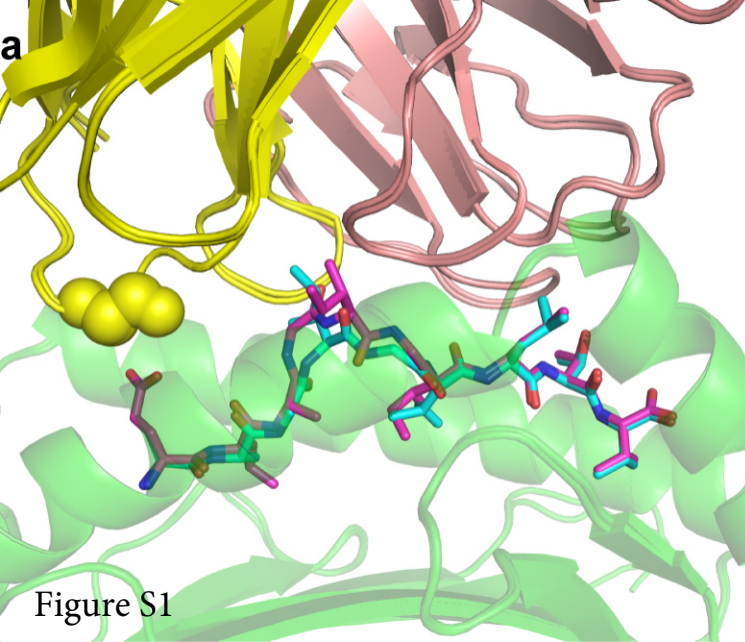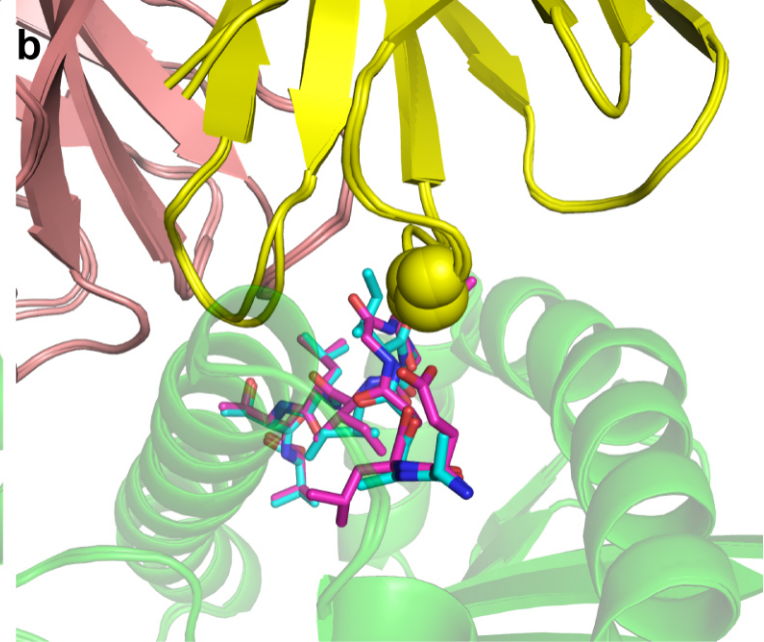

Supplement: Figure S1 — Structural variability of nonameric (AAG; cyan) and decameric (ELA; magenta) MART-1 peptides bound to wild-type DMF5 (from wild-type complex structures, PDB IDs 3QDJ and 3QDG). MHC and TCR colors are as in Figure 4; DMF5 residue αG28 is shown as spheres for reference. (PDF) [file pcbi.1003478.s001.pdf]

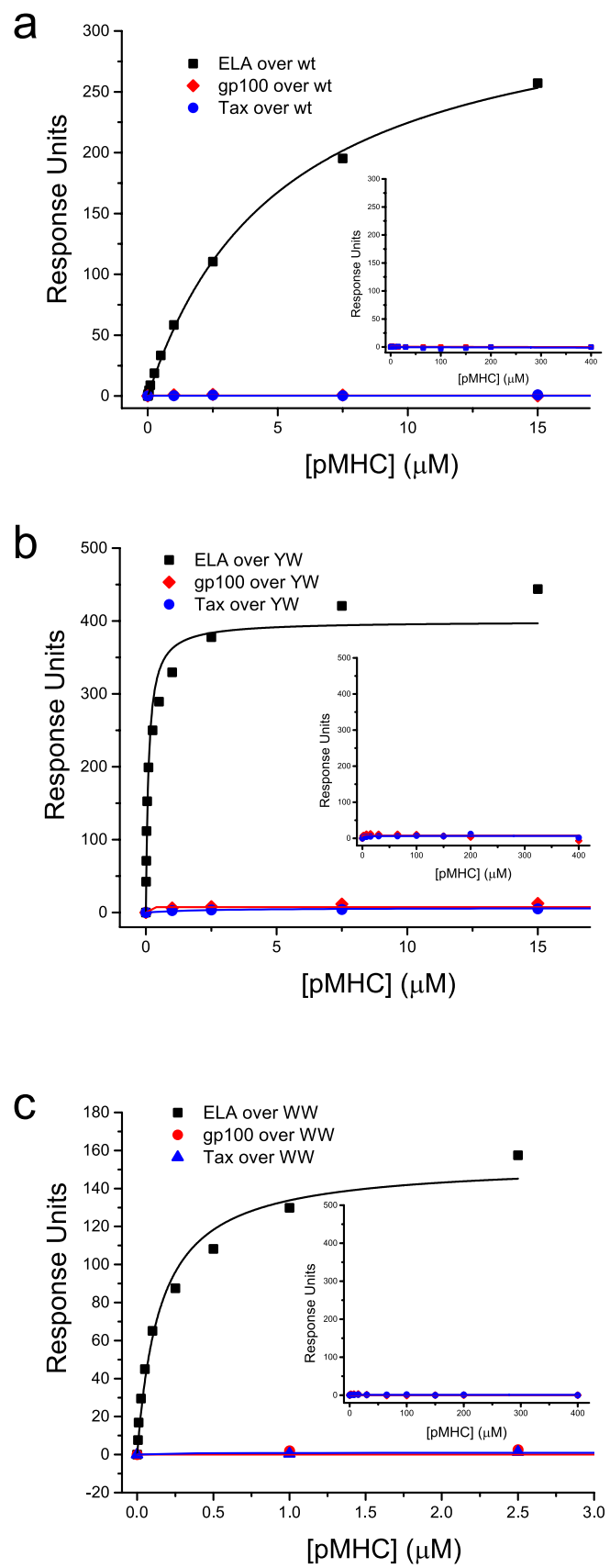

Figure S2

Supplement: Figure S2 — The high affinity DMF5 variants show no recognition of the Tax11–19 or gp100209(2M)-217 peptide/HLA-A2 complexes. a) Injections over a wild-type DMF5 surface. The main response shows injections of MART-126(27L)-35/HLA-A2, with the binding response indicated. The inset shows injections of gp100/HLA-A2 and Tax/HLA-A2 over the same surface, with no response at concentrations as high as 400 mM. b) Injections over a high affinity YW DMF5 surface. Injected pMHC is as in panel a. c) Injections over a high affinity WW DMF5 surface. Injected pMHC is as in panel a. (PDF) [file pcbi.1003478.s002.pdf]

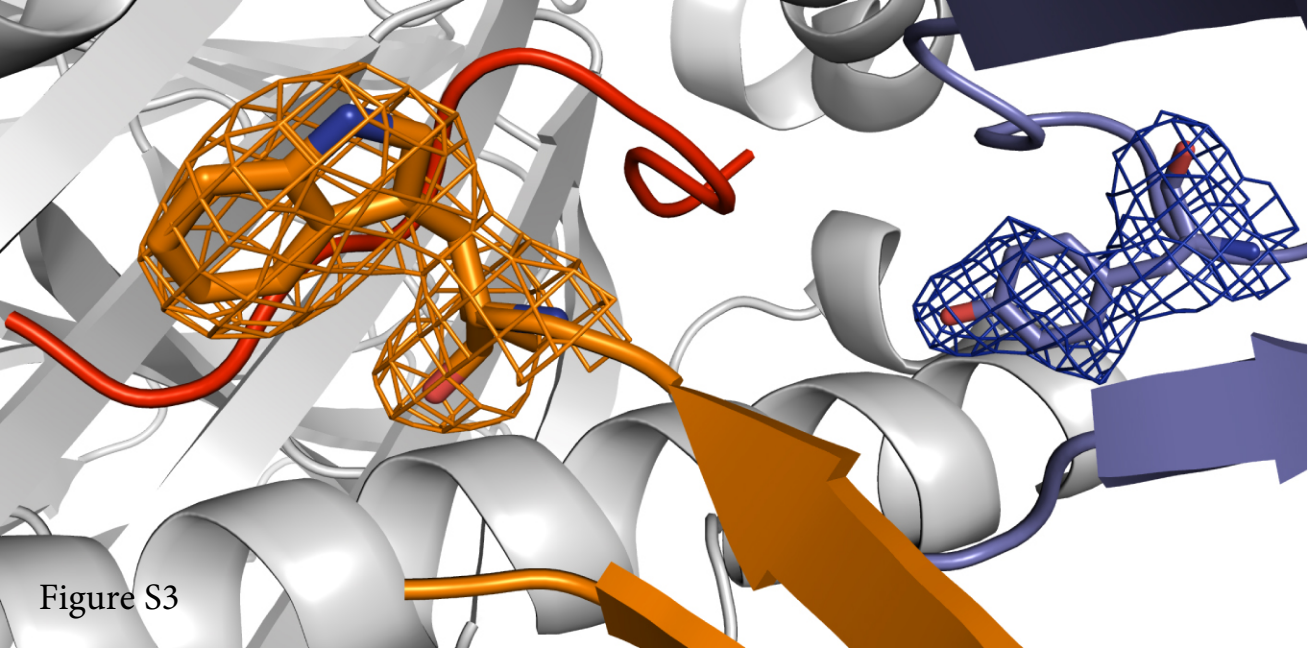

Figure S3

Supplement: Figure S3 — Electron density for βW98 (gold) and αY26 (purple) in the YW-ELA/HLA-A2 crystal structure contoured at 1σ calculated from an unbiased, iterative-build OMIT map. The density shows the clear, unambiguous positioning of the two mutated residues. (PDF) [file pcbi.1003478.s003.pdf]

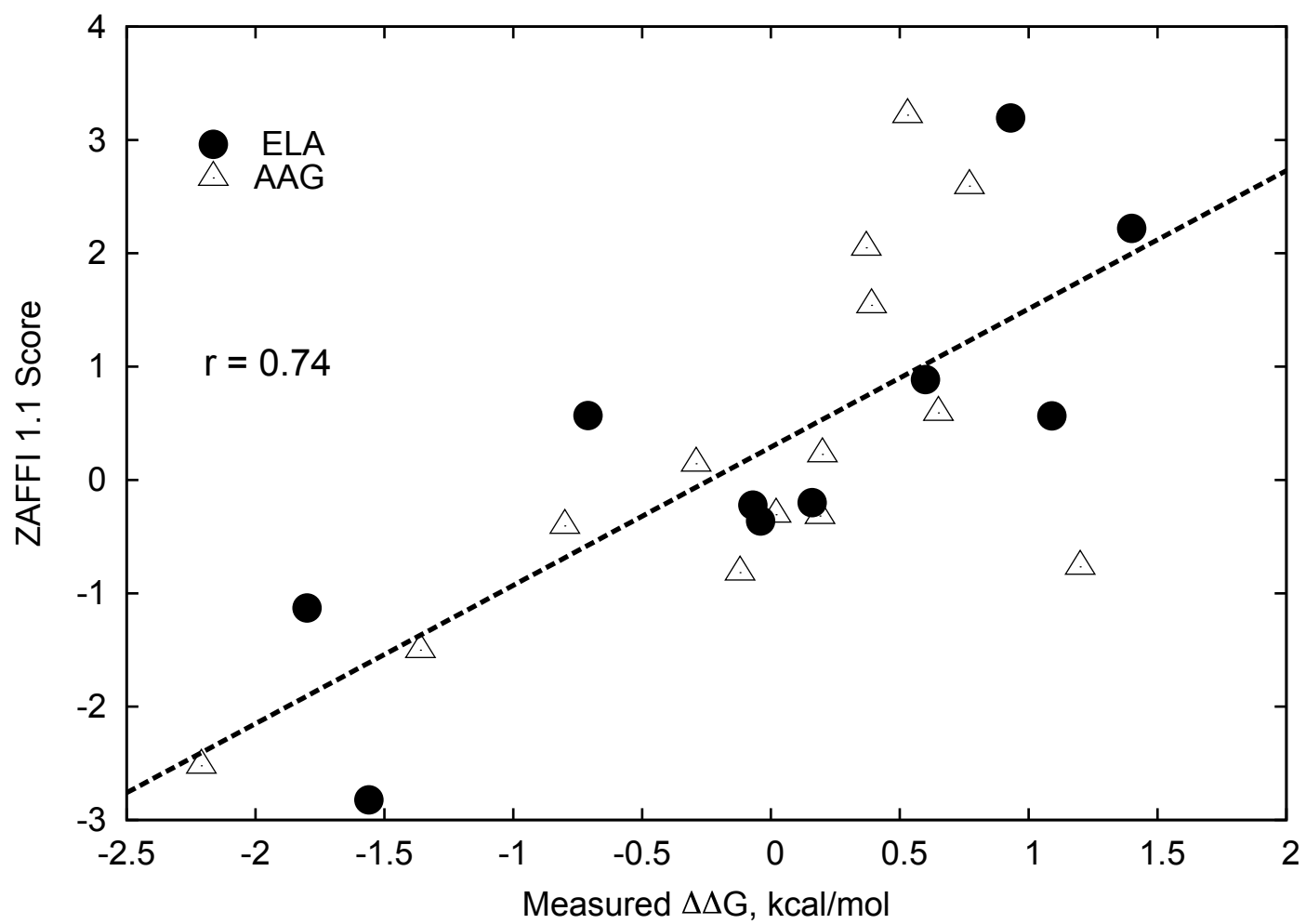

Figure S4

Supplement: Figure S4 — Predictions from ZAFFI 1.1 compared with measured ΔΔGs for DMF5 point mutants binding to ELA/HLA-A2 (solid circles) and AAG/HLA-A2 (empty triangles). Best fit line and correlation are given; the four true negative outlier points omitted from Figure 3 are omitted here as well. (PDF) [file pcbi.1003478.s004.pdf]

Figure S5

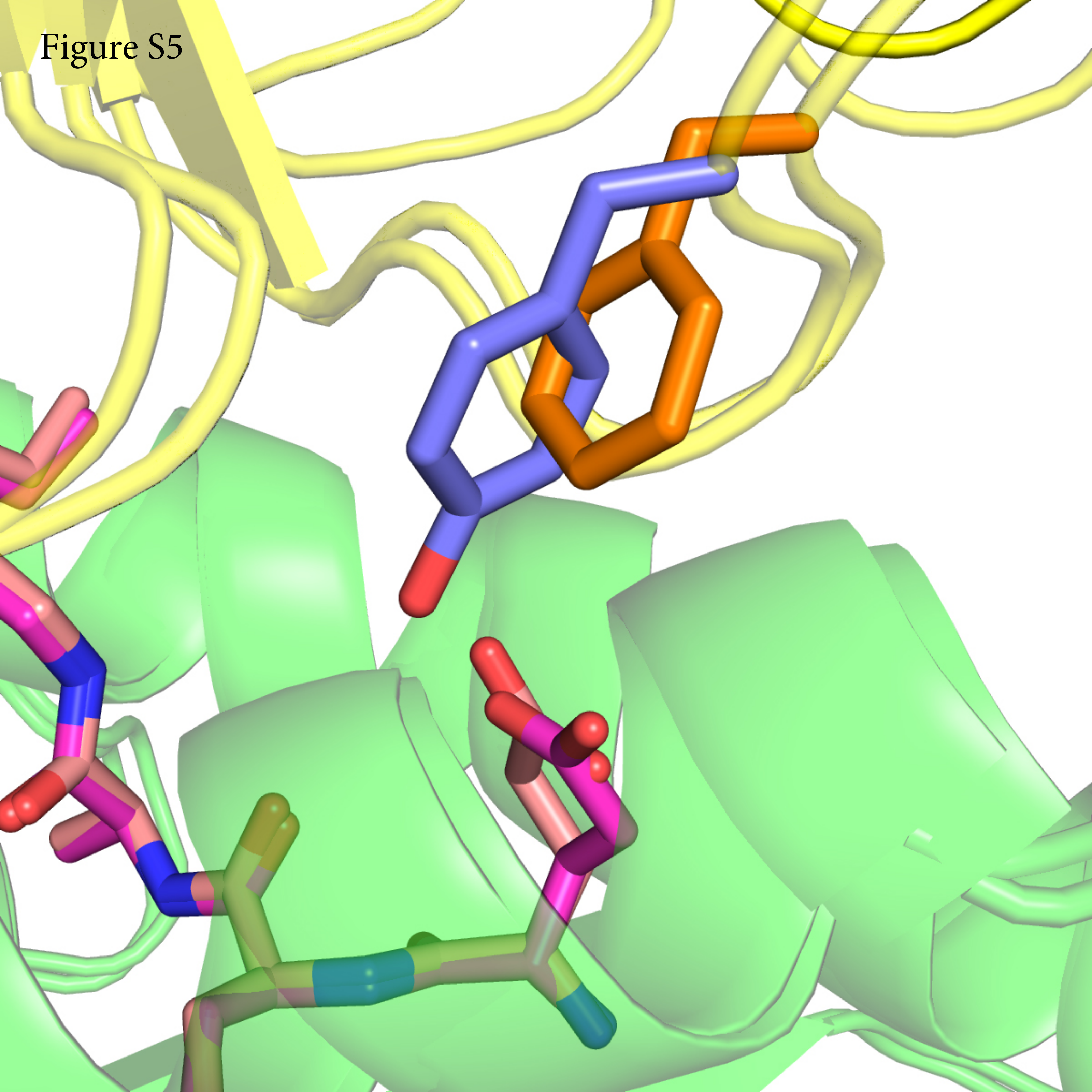

Supplement: Figure S5 — Comparison of mutant TCR αD26Y residue in the YW-ELA/HLA-A2 complex with the corresponding mutant position (αD27F) in the α24β17-ELA/HLA-A2 complex. Complexes were superposed by fitting pMHC backbone atoms. The mutant αD27F is shown in orange sticks, ELA peptide from α24β17-ELA/HLA-A2 in pink sticks, and all other colors are as in Figure 4. (PDF) [file pcbi.1003478.s005.pdf]
