# Supplementary material for: Computational Design of the Affinity and Specificity of a Therapeutic T Cell Receptor
Source: PLoS Comput Biol. 2014 Feb 13;10(2):e1003478. doi: 10.1371/journal.pcbi.1003478 (PMC3923660; doi:10.1371/journal.pcbi.1003478)
Supplement: Table S1 — DMF5 mutant predictive performance for additional tested packing protocols. (PDF) [file pcbi.1003478.s006.pdf]

Table S1. DMF5 mutant correlations for additional tested packing protocols.

**Packing**[1]	**Rosetta Correlation**	**ZAFFI Correlation**
Repack	0.42	0.41
Min_rb	0.57	0.51
Min_bb_chi	0.47	0.47
Min_all	0.52	0.52
NoMin_h2o	0.59	0.59
NoMin_h2o_mut	0.59	0.59

[1]Packing protocol in Rosetta: Repack = neighbor residues repacked; Min_rb = rigid-body protein minimization; Min_bb_chi = backbone and side chain minimization of all residues; Min_all = rigid-body minimization plus interface backbone and side chain minimization; NoMin_h2o = only mutant side chains were packed, water molecules from wild-type complex structures were used; NoMin_h2o_mut = only mutant side chains were packed, water molecules from YW/ELA/A2 structure were fitted to the wild-type complex structure and used for ELA mutant predictions (wild-type complex waters were used for AAG).
