# Supplementary material for: Computational Design of the Affinity and Specificity of a Therapeutic T Cell Receptor
Source: PLoS Comput Biol. 2014 Feb 13;10(2):e1003478. doi: 10.1371/journal.pcbi.1003478 (PMC3923660; doi:10.1371/journal.pcbi.1003478)
Supplement: Table S2 — X-ray data collection and refinement statistics for the crystal structure of the DMF5 αD26Y/βL98W - ELA/HLA-A2 complex. (PDF) [file pcbi.1003478.s007.pdf]

Data Collection	
Source	22ID
Space group	C121
Cell dimensions:	
a, b, c (Å)	227.0, 49.3, 92.9
α, β, γ (°)	90.0, 94.8, 90.0
Resolution (Å)	20 – 2.55 (2.64 – 2.55)
R_{merge}	0.09 (0.44)
I/σI	11.7 (2.0)
Completeness (%)	95.1 (83.3)
Redundancy	3.8 (2.9)
**Refinement**	
Resolution (Å)	20 – 2.56
No. reflections	31,818
R_{work}/R_{free}	0.23/0.27
No. atoms:	
Protein	6603
Water	12
B-factors:	
Protein	47.9
Water	23.1
RMSD from ideality:	
Bond length (Å)	0.009
Bond angle (°)	1.151
Ramachandran plot:	
Favored	98%
Allowed	99%
PDB entry	4L3E

Table S2. X-ray data collection and refinement statistics for the crystal structure of the DMF5 αD26Y/βL98W - ELA/HLA-A2 complex.
