# Supplementary material for: Computational Design of the Affinity and Specificity of a Therapeutic T Cell Receptor
Source: PLoS Comput Biol. 2014 Feb 13;10(2):e1003478. doi: 10.1371/journal.pcbi.1003478 (PMC3923660; doi:10.1371/journal.pcbi.1003478)
Supplement: Table S3 — Contacts between mutant DMF5 residues and ELA/HLA-A2. (PDF) [file pcbi.1003478.s008.pdf]

Table S3. Contacts between mutant DMF5 residues and ELA/HLA-A2.

TCR residue	pMHC Residue	Contact Type	wild-type[1]
Tyr-26^{OH}	**pGlu-1$^{O\varepsilon 2}$**	H bond	None
Tyr-26^{OH}	**pGlu-1$^{O\varepsilon 1}$, Lys-66^{Nz}**	water-mediated H bond	None
Tyr-26	**Glu-58, Gly-62, Glu-63, Lys-66**	van der Waals (11)	0
Trp-98	pLeu-8, Ala-150	van der Waals (7)	3

Peptide residues are preceded with a “p”, and peptide and MHC residues not contacted by the wild-type residues are in bold. Hydrogen bonding atoms are noted with a superscript. van der Waals contacts were calculated with a 4.0 Å distance cutoff; numbers in parentheses denote the number of van der Waals contacts between TCR and pMHC atoms.

[1]Whether the hydrogen bond is present for the wild-type TCR residue, or the number of van der Waals contacts involving the wild-type residue in the ELA/HLA-A2 complex (PDB ID 3QDG).
