# Supplementary material for: Computational Design of the Affinity and Specificity of a Therapeutic T Cell Receptor
Source: PLoS Comput Biol. 2014 Feb 13;10(2):e1003478. doi: 10.1371/journal.pcbi.1003478 (PMC3923660; doi:10.1371/journal.pcbi.1003478)
Supplement: Table S4 — Correlations with measured values and corresponding p-values for HB36 and HB80 mutants. (PDF) [file pcbi.1003478.s009.pdf]

Table S4. Correlations with measured values and corresponding p-values for HB36 and HB80 mutants.

Function	Packing	HB36 Corr	HB36 P-value	HB80 Corr	HB80 P-value	HB36 Kendall[1]	HB80 Kendall[1]
Rosetta	NoMin	0.31	7.1E-08	0.33	9.5E-09	0.21	0.31
ZAFFI	NoMin	0.32	4.0E-08	0.33	7.6E-09	0.16	0.23
Rosetta	Min	0.41	9.5E-13	0.30	2.7E-07	0.28	0.19
ZAFFI	Min	0.34	4.3E-09	0.37	1.1E-10	0.17	0.22
ZAFFI 1.1	NoMin	0.36	2.1E-10	0.50	< 2.2E-16	0.22	0.38

HB36 and HB80 each represent 285 interface point mutants of proteins designed to bind influenza hemagglutinin [1]. P-values were calculated for the Pearson correlations (“Corr”).

[1]Kendall tau rank correlation coefficient

1. Moretti R, Fleishman SJ, Agius R, Torchala M, Bates PA, et al. (2013) Community-wide evaluation of methods for predicting the effect of mutations on protein-protein interactions. Proteins 81: 1980-1987.
